# Effects of Two Commonly Found Strains of Influenza A Virus on Developing Dopaminergic Neurons, in Relation to the Pathophysiology of Schizophrenia

**DOI:** 10.1371/journal.pone.0051068

**Published:** 2012-12-10

**Authors:** Fernando Landreau, Pablo Galeano, Laura R. Caltana, Luis Masciotra, Agustín Chertcoff, A. Pontoriero, Elsa Baumeister, Marcela Amoroso, Herminia A. Brusco, Mónica I. Tous, Vilma L. Savy, María del Rosario Lores Arnaiz, Gabriel A. de Erausquin

**Affiliations:** 1 Cultivo de Tejidos, Departamento Virología, Instituto Nacional de Enfermedades Infecciosas, ANLIS “Dr Carlos G. Malbran”, Buenos Aires, Argentina; 2 Laboratorio de Citoarquitectura y Plasticidad Neuronal, Instituto de Investigaciones “Prof. Dr. Alberto C. Taquini” (ININCA), Facultad de Medicina, Universidad de Buenos Aires, Buenos Aires, Argentina; 3 Instituto de Biología Celular y Neurociencia “Profesor E. De Robertis”, Facultad de Medicina, Universidad de Buenos Aires, Buenos Aires, Argentina; 4 Bioterio Central, Instituto Nacional de Producción de Biológicos, ANLIS “Dr Carlos G. Malbran”, Buenos Aires, Argentina; 5 Virus Respiratorios, Departamento Virología, Instituto Nacional de Enfermedades Infecciosas, ANLIS “Dr Carlos G. Malbran”, Buenos Aires, Argentina; 6 Microscopía Electrónica, Departamento Virología, Instituto Nacional de Enfermedades Infecciosas, ANLIS “Dr Carlos G. Malbran”, Buenos Aires, Argentina; 7 Roskamp Laboratory of Brain Development, Modulation and Repair, Department of Psychiatry and Neurosciences, University of South Florida, Tampa, Florida, United States of America; 8 Facultad de Psicología, Universidad de Buenos Aires, Buenos Aires, Argentina; Emory University, United States of America

## Abstract

Influenza virus (InfV) infection during pregnancy is a known risk factor for neurodevelopment abnormalities in the offspring, including the risk of schizophrenia, and has been shown to result in an abnormal behavioral phenotype in mice. However, previous reports have concentrated on neuroadapted influenza strains, whereas increased schizophrenia risk is associated with common respiratory InfV. In addition, no specific mechanism has been proposed for the actions of maternal infection on the developing brain that could account for schizophrenia risk. We identified two common isolates from the community with antigenic configurations H3N2 and H1N1 and compared their effects on developing brain with a mouse modified-strain A/WSN/33 specifically on the developing of dopaminergic neurons. We found that H1N1 InfV have high affinity for dopaminergic neurons in vitro, leading to nuclear factor kappa B activation and apoptosis. Furthermore, prenatal infection of mothers with the same strains results in loss of dopaminergic neurons in the offspring, and in an abnormal behavioral phenotype. We propose that the well-known contribution of InfV to risk of schizophrenia during development may involve a similar specific mechanism and discuss evidence from the literature in relation to this hypothesis.

## Introduction

Influenza virus (InfV) infection during pregnancy is a known risk factor for neurodevelopment in the offspring, increasing the risk of schizophrenia [1]–[6] and autism [7]–[9], and resulting in an abnormal behavioral phenotype in mice [10]. The peak of this effect occurs during the 6th month of intrauterine development in humans [7], and it has been shown to correlate with serologically proven infection in the mother [1]. However, the relationship between infection and disease remains controversial [11]–[14].

Experimental infection of pregnant mice with InfV results in pathological and physiological changes in the brain of offspring that resemble those observed in schizophrenia [10], [15]–[19]. Such effects of InfV on neurodevelopment appear to be at least in part mediated through immune mechanisms [10], [20]–[22], but infection of pregnant mice with InfV may also result in persistent expression of virus in fetal brain [23].

Regardless of the molecular mechanism by which infection results in neurodevelopmental changes, deviant behavior in adult offspring should have a correlate in structural or functional changes in brain circuitry, and there are reasons to consider the dopaminergic system as a potential target. Indeed, intracerebral inoculation of InfV in adult rodents results in viral antigen accumulation in dopaminergic neurons [24]–[25], and a similar preference for dopaminergic neurons can be demonstrated in primary neuronal cultures [26]. Direct stereotaxic introduction of InfV in mice olfactory bulb leads to apoptosis of infected neurons [27]–[28]. Yet, these findings refer to neuroadapted strains of InfV, whereas the most common viral epidemics of InfV A are rarely associated with encephalitis [25]. InfV A viruses are RNA viruses characterized by two surface antigens: hemagglutinin (H) and neuraminidase (N) [25]. Variations in these two antigens occur independently from strain to strain, and can involve major (shift) or minor (drift) alterations and at least in mice neurovirulence is determined by the plasminogen binding activity of neuraminidase [29]. Spread of a virus and tissue tropism may depend largely on the proper match between cleavability of viral glycoproteins by tissue endopeptidases and availability of proteases in the host, the latter being the limiting factor [25]. Once again, a virus activating protease, blood clotting factor Xa (FXa), is selectively present in brainstem neurons, including dopaminergic neurons in the substantia nigra pars compacta, and in white matter microglia [25], [30].

Is there any evidence that susceptibility of dopaminergic neurons to InfV A infection may modify risk of schizophrenia? First, the observation that parkinsonism can occur as a trait associated with schizophrenia suggests a dopaminergic deficit [31]–[35]. Functional imaging and postmortem studies support a loss of dopaminergic function in schizophrenia [36]–[42] and the patients’ impairment in executive function and working memory correlates with low dopamine availability in the prefrontal cortex [41]–[42]. On the other hand, psychotic symptoms correlate with excessive release of dopamine in basal ganglia [43]. Therefore, the available evidence points to the simultaneous lack and excess in dopaminergic function albeit in different brain circuits; in rodents, prenatal damage to a subpopulation of dopaminergic neurons projecting from the midbrain to the prefrontal cortex has been shown to lead over time to a maladaptive, compensatory increase in mesolimbic dopaminergic activity upon the arrival of puberty [43]–[45].

Thus, we hypothesized that maternal infection with InfV during the critical period of risk for schizophrenia (the equivalent of the human second trimester, or embryonic days 11–16 in rodents), should result in preferential damage to developing dopaminergic neurons in the brainstem of offspring. We further hypothesized that such damage depends on the antigenic configuration of circulating InfV. In the present work we evaluated both of these hypotheses in primary cultures of ventral mesencephalon and in a mouse model of prenatal infection with circulating InfV strains.

## Materials and Methods

### Primary Cultures of Rat Mesencephalon

All animal work was conducted according to relevant national and international guidelines and approved by the Animal Studies Committee of ANLIS Carlos G Malbran (Resolución 20/4/2004). Rat embryos were recovered at day 14 from timed pregant Wistar rats, and the ventral midbrain was dissected, mechanically dissociated, and plated on polyethylenimine (1 mg/ml)-coated culture wells, in DMEM/F12 medium, supplemented with 5% horse serum (US) and *2.5%* fetal calf serum (FCS), at a density of 1×10^6^ cells per 6-mm-diameter well. After 2 days in culture FCS content was reduced to 0.5% to prevent astrocyte proliferation; medium was changed daily. Cultures are maintained for 7–11 days in vitro. Dopaminergic neurons can be identified after fixation by immunohistochemistry for tyrosine hydroxylase (TH). This is the accepted standard methodology to prepare dopaminergic neurons in culture, in part because at this developmental age, dopaminergic neurons account for 10–20% of the total number of neurons, express specific phenotypic markers and functional glutamate receptors, and release and uptake dopamine [46]–[49].

### MDCK Cells

Replication of viruses under liquid medium and plaque determinations are performed in MDCK cells (passage 59) obtained from the Centers for Disease Control and Prevention (CDC, Atlanta, GA).

### Viral Strains

We identified two common isolates from community samples of Buenos Aires from the Instituto Malbrán, with antigenic configurations H3N2 and H1N1. The results of characterization with ferret antisera are: A/New Caledonia/20/99-like (H1N1) (A/NC-L/99), and A/Sydney/5/97-like (H3N2) (A/Sy-L/97). Isolates were amplified in MDCK cells and frozen in lots of 100 cryotubes. We used a well characterized neurovirulent strain obtained from the CDC (Atlanta, GA) (A/WSN/33) as a positive control for toxicity to dopaminergic neurons [25] and behavioral abnormalities in the offspring of infected pregnant mice [10].

### Viral Quantitation

Titration of plaque forming units (PFU) was performed in MDCK cells and normalized for each lot to:

Lot 1 A/Sy-L/97 (H3N2) 3,000 PFU/ml.

Lot 1 A/NC-L/99 (H1N1) 3,000 PFU/ml.

Lot 1 A/WSN/33 (H1N1) 3,000 PFU/ml.

### Influenza a Genome Amplification

Viral RNA was purified from selected samples by QIAamp®Viral RNA Mini Kit.

### Reverse Transcription-polymerase Chain Reaction (RT-PCR)

RT PCR was used to amplify regions of the protein coding domains of viral RNA segment 7 to obtain finally an amplicon of 560 bp, using the QIAGEN®One Step RT-PCR Kit.

### Influenza a Antigen Detection

The antigen detection was performed by Indirect immunofluorescent assay (IFA) using the standard antibody for surveillance recommended by CDC (Atlanta, GA), namely mouse monoclonal anti influenza a nucleoprotein (immunized with Influenza A/Puerto Rico/8/34 (H1N1) and A/Bangkok/1/79 (H3N2) viruses), from spleen cells from BALB/c mice fused with cells of the P3 Ag8.653 mouse myeloma cell line (AbD Serotec, USA).

### In vitro Infection

**Figure 1:**
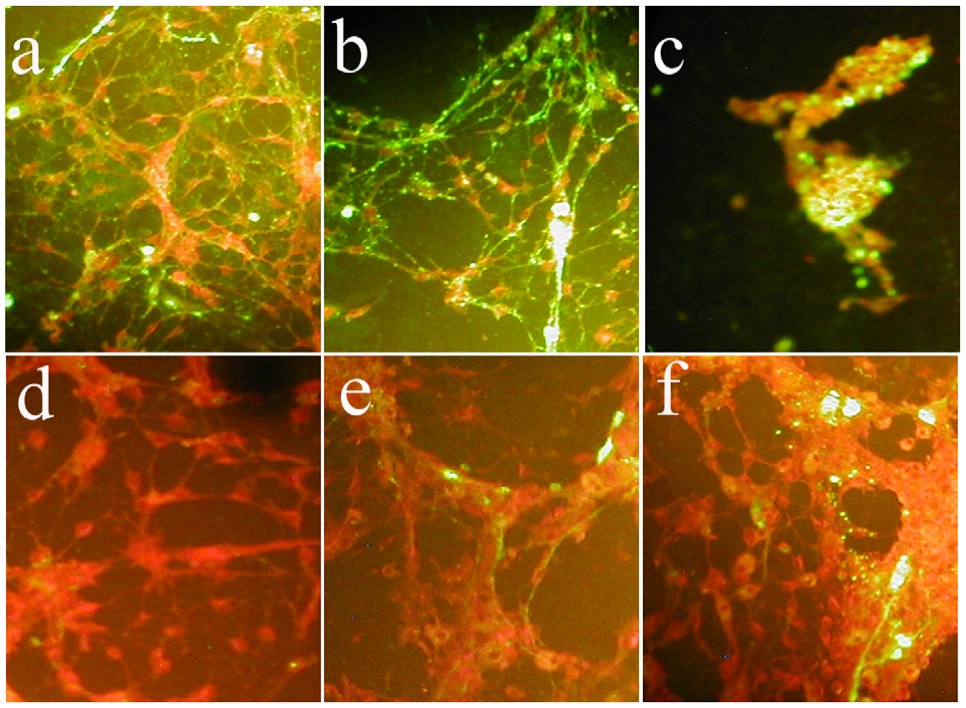
Indirect immunofluorescence for Influenza virus in primary mesencephalic cultures. Culture dishes infected with New Caledonia-like (H1N1) (panels a–c) or Syndey-like (H2N3) strains (panels d–f), at 24 (a, d), 48 (b, e) and 72 hours (c, f) post inoculation. The two community isolates differed from each in that the New Caledonia-like strain (A/NC-L/99) showed greater affinity at earlier times and more toxic than Sydney-like (A/Sy-L/97) virus.

For neurotropism experiments we inoculated 50 µl of viral suspension (150 PFU/well) in primary cultures of rat mesencephalon, and examined the presence of viral infected cells at 24 h, 48 h and 72 h (after fixation with formalin), by indirect immunofluorescence with monoclonal anti-InfV A antibodies (CDC, Atlanta, GA) followed by fluorescein-conjugated anti mouse (Light Diagnostics) (Figure 1). 50% Tissue Culture Infective Dose (TCID50) was calculated for each viral strain using the Reed and Muench Calculator [50] (Brett D. Lindenbach, 2008).

### In vivo Infection

BALB-C mice were obtained from the Bioterio Central of the Instituto Nacional de Producción de Biológicos. Because infection entails discomfort to the animals, we minimized it by ensuring adequate hydration, temperature control, and cage conditions according to strict guidelines (Instituto Malbrán/CDC). Intranasal inoculation with 30 µl (3.000 PFU/ml of InfV, 90 PFU/mouse) strains or vehicle (PBS, pH 7.4) was performed at embrionic day 9–11 on the assumption that viremia peaks (and most likely invades CNS) three days later (and therefore ∼ embryonic day 14). Post-infection all animals were individually kept in filtered cages with ad lib access to food and water, at 25°C. Seroconversion was established by inhibition of hemagglutination of turkey red blood cells at day 14 following infection. We calculated the 50% infective dosing by measuring change in lung weights post infection in lots of 5 animals for each strain and for each of four dilutions. ID50 (expressed as dilutions of a normalized hemagglutinin unit) for each strain were: A/Sy-L/97 = 1.07E-02, A/NC-L/99 = 1.41E-02, and A/WSN/33 = 2.49E-02. We did not find lethal infections at any of the used viral loads and therefore LD50 could not be estimated for any strain. Institutional and ethical committee approval for this protocols was obtained at both centers. ID50 was calculated for each viral strain using the Reed and Muench Calculator [50] (www.med.yale.edu/micropath/pdf/Infectivity%20calculator.xls, Brett D. Lindenbach, 2008).

### Behavioral Studies

Pups from infected mothers were examined in a behavioral test battery including elevated plus maze, open field, novel object, and spontaneous activity before puberty (30 days) or in adulthood (90 days). In the latter we also carried out an object recognition task (90 days). Behavioral tests were carried out in an experimental room evenly illuminated provided with white noise. Animals were handled for 5 min daily for 3 days prior to testing. Sessions were recorded and later analyzed using a computerized video-tracking system (Ethovision XT, version 5, Noldus Information Technology, Wageningen, Netherlands) or ethological observation software (JWatcher V1.0).

#### Elevated plus maze

Elevated plus maze testing was carried out in a standard mice apparatus elevated 37.5 cm from the floor. At designated times animals were placed onto the centrdal platform facing an open arm and allowed to freely explore the maze for 5 min. After each session the apparatus was cleaned. An arm entry was counted when all four paws were placed into an arm.

#### Open field

Animals were placed in the center of a standard open field apparatus and total distance moved, number of rearings and time spent in the central area were recorded for 20 minutes. A mouse was considered to be into the central area (arbitrarily defined as a square of 30×30 cm) when its four paws were in it. The apparatus was cleaned between sessions.

#### Novel object

Upon completion of the 20-min open field session, a novel object (a metal cup), was placed in approximately 20 cm away from the mouse and the latency to contact the object and time spent in contact with it were recorded for 10 min.

#### Spontaneous activity

Two days after the open field and novel object tests, the spontaneous locomotor activity was monitored using a webcam and quantified using a simple criteria for number of squares crossed per unit time (24 h).

#### Object recognition task

For 3 consecutive days prior to this task mice were handled once a day for 5 min and placed 10 min in the open field to allow habituation. On the fourth day each mouse was observed during two 5 min trials separated by an interval of 1 h during which animals were returned to their cage. In the sample trial (T1) mice were faced with two identical objects placed in a symmetrical position and the time exploring each object was recorded. In the retention trial (T2) mice one of the two objects was replaced by a novel, non-familiar object and the time exploring each was recorded. Sets composed of three copies of the same object were used to prevent odor cues and all combinations and location of objects were used to prevent bias due to preference for a particular object or location. Exploration time was computed when the snout pointed to the object at a distance ≤2 cm.

### Histological Studies

After behavioral testing mice were sacrificed and fixed, brain sections (15 µm) were obtained in stereological series (every 8^th^ section), and processed for histopathological analysis that included gross morphology with Nissl staining, stereological quantitation of immunohistochemistry for dopaminergic neurons and reactive astroglia in the substantia nigra and ventral tegmental area, and electron microscopy of dopaminergic neurons stained with tyrosine hydroxylase. Post-fixed brains stored in 1× PBS containing 0.05% sodium azide were embedded in 3% low-melting agarose, sectioned cornally (40 µm) on a vibratome (Leica VT1200) and stored in cryoprotectant (30% glycerol, 30% ethylene glycol in 1× PBS) at −20°C until processing. Immunohistochemistry was performed on every 6th section. For immunohistochemistry sections were washed in PBS endogenous peroxidases were blocked by incubation in 3% H2O2, and blocking of non-specific binding was achieved by incubating in PBS containing 0.25% (w/v) Triton-X 100 and 10% normal serum. Sections were incubated overnight at 4°C with one of the following primary antibodies: Tyrosine Hydroxylase (TH, Pelfreeze Biologicals, Rogers, AR; or Boehringer GmbH, Mannheim, Germany), Nuclear Factor kappa B (NFkBp65, Abcam, Cambridge, MA), Anti-GFAP antibody - Astrocyte Marker (ab4674, Abcam, Cambridge, MA) and Anti-InfV A antibodies provided by the Instituto Malbrán (Buenos Aires, Argentina). For microglia activation we used lectin staining (IsoB4) and antibody staining (ED-1). Following washing, sections were then incubated with appropriate biotinylated secondary antibody (Jackson ImmunoResearch Laboratories, West Grove, PA) for 1 hr followed by incubation with avidin-biotin-peroxidase complex (ABC Elite Kit; Vector Laboratories) detection system with diaminobenzidine (Ultratech HRP Streptavidin-biotin Universal Detection System and DAB Chromogen Kit, Inmunotech Co, Marseille, France). For immunofluorescence labeling, sections were stained with streptavidin conjugated Alexa Fluor 568 (working solution 1–5 µg/ml; Molecular Probes, Eugene, OR). For detection and quantification of apoptosis (programmed cell death) at single cell level, we used a protocol based on labeling of DNA strand breaks (TUNEL technology) carried out following manufacturer’s recommendations (DeadEnd™ colorimetric TUNEL System, Promega, Madison, WI).

### Electron Microscopy

Embryonic brains 3 days after maternal infection were fixed with 3% v/v glutaraldehyde in PBS overnight, washed, postfixed in 1.5% w/v osmium tetroxide in the same buffer for 2 hs, contrasted with 2% w/v acuose uranyl acetate, and embedded in Fluka Poly-Bed 812 (Sigma-Aldrich, St Louis, MO). Ultrathin sections (90–100 nm) were collected in cupper grids and stained with uranyl acetate and Reynolds solution, and imaged using a Philips EM300 transmission electron microscope.

### Data Analysis

All experiments were carried out in triplicates. For stereology, cells were counted using every 8^th^ consecutive section, 400 µm apart from each other, throughout the entire ventral mesencephalon (substantia nigra compacta and ventral tegmental area) using a Nikon 80i Eclipse microscope (Tokyo, Japan) equiped with Stereologer software (SRC, Tampa, FL) and a motorized stage. Digital photographs were taken using a Zeiss camera with AxioVision software (Carl Zeiss Microscopy GmbH, Göttingen, Germany). Digital images were then processed with ImageJ (NIH). The cross sectional area of each section was determined with Stereologer and the data were expressed as number of cells per hemibrain. Statistical analyses were performed with SPSS software (IBM Corporation, Somers, NY). Data are presented as mean ± standard error of the mean (SEM). Comparisons among groups were performed using a one or two way ANOVA as indicated, followed by a Kruskal–Wallis post hoc test as appropriate. For significant differences α was set at 0.05. For multivariate analysis of behavioral performance, discriminant analysis was performed using prenatal exposure as the classification criterion (SPSS, IBM Corporation, Somers, NY).

## Results

### Community Strains of InfV have High Affinity for Mesencephalic Cultures in vitro and Result in Neuronal Death

Epidemiological reports show an association between respiratory infection by InfV A in pregnancy and risk of schizophrenia to the offspring; we investigated the effects of common respiratory InfV A strains on development of dopaminergic neurons in primary mesencephalic cultures to test the hypothesis that InfV A could mediate its effects on schizophrenia risk through developmental damage to specific dopaminergic projections [43]–[45]. We first assessed the affinity of viral strains for neurons with IFA for InfV A community isolates using normalized viral loads. Both strains infected neurons effectively, but A/NC-L/99 had greater infectivity at earlier times than A/Sy-L/97 (Figure 1). This result was consistent with the calculated TCID50 which was lowest for A/Sy-L/97 (TCID50 for A/WSN/33 = 1.43E-03, for A/NC-L/99 = 3.16E-03, and for A/SY-L/97 = 3.98E-02). Dopaminergic neurons were identified by TH immunoreactivity, since loss of TH staining correlates tightly with cell death in these cultures [48], [49], [51]–[53].

**Figure 2:**
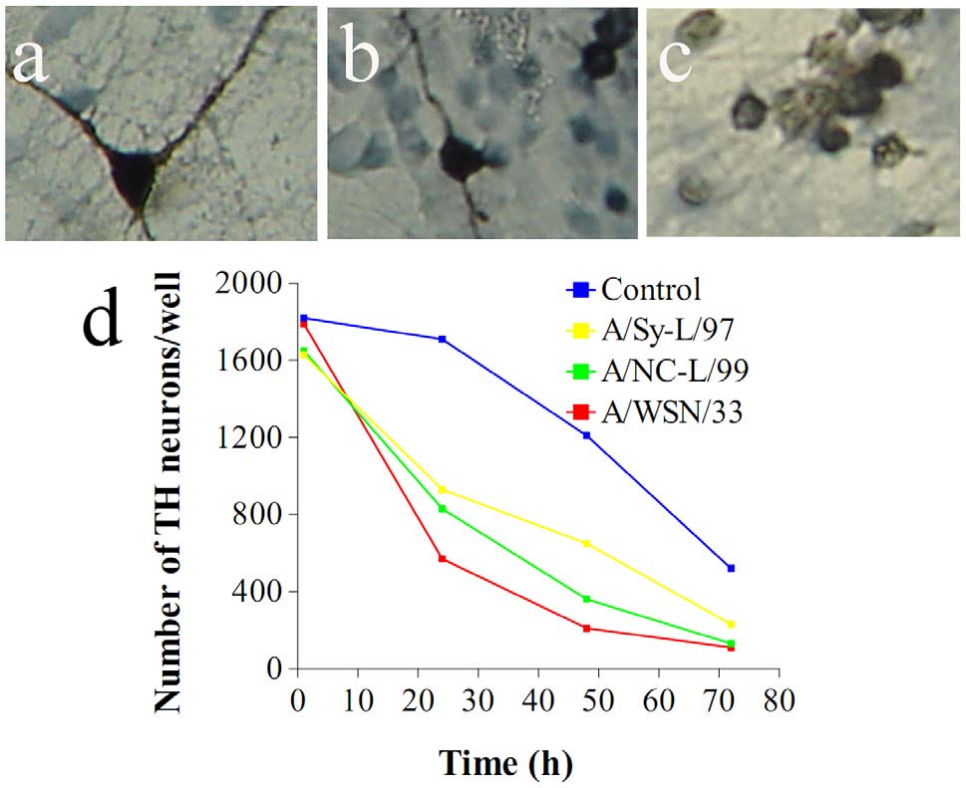
Influenza virus promotes neuronal death of dopaminergic neurons in vitro. Panels (a–c) display characteristic morphology changes of TH stained neurons at baseline (a), 48 h (b) and 72 h (c) post infection; dopaminergic neurons are clearly damaged by the virus, with loss of dendrites and vacuolation of the cytosol. Time curves of TH stained neuronal counts for each viral strain regarding of their morphological state, and therefore probably representing an overestimation of surviving neurons. The A/WSN/33 strain was most toxic at 24 and 48 h. Points represent means of 6–8 independent experiments (two wells per experiment). Standard errors are two small to be displayed on this scale (range 11 to 86). Comparisons were performed by two way ANOVA (treatment and time). ** p<0.001 compared to vehicle.

**Figure 3:**
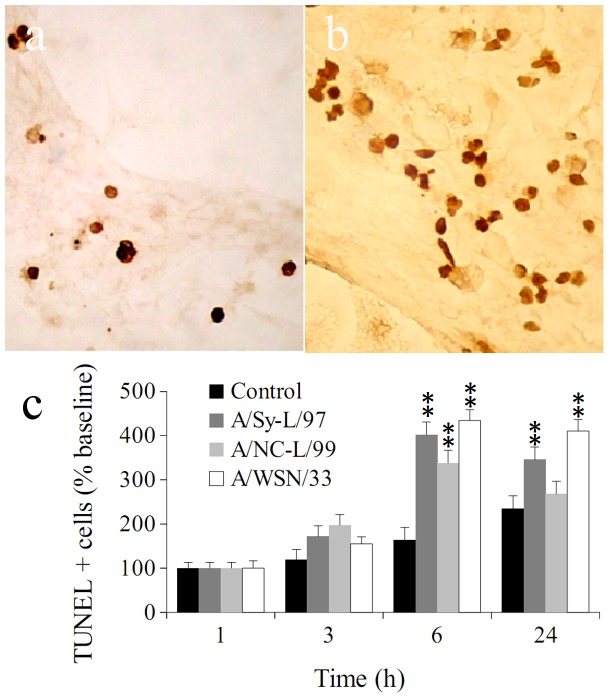
Influenza infection causes neuronal apoptosis in vitro. Panels a and b show TUNEL staining in primary cultures following inoculation of cultures (11 day in vitro) with either PBS solution (a) or A/WSN/33 (d) at 200X. Cultures were fixed, stained for TUNEL and counted at times specified on the x axis of panel (c). Cell counts bars represent means of 6–8 independent experiments (two wells per experiment). Error bars are SEM. Comparisons were performed by two way ANOVA (treatment and time). ** p<0.001 compared to control (PBS solution).

Because control cultures undergo progressive attrition of neurons in serum-free conditions we compared the effect of infection to spontaneous neuronal loss over time in PBS inoculated wells (Figure 2A). Following infection, TH stained neurons undergo rapid loss of dendrites, cytosolic vacuolation and nuclear picnosis (Figure 2C–E). Yet, since TH immunostaining does not directly establish the presence of cell death, we carried out TUNEL staining to detect DNA fragmentation. As expected, in control cultures changed to serum free medium attrition by apoptosis was detected by 24 h. By contrast, cultures inoculated with InfV strains underwent significant apoptosis 6 hours post-infection. Notably, apoptosis was most apparent in cultures exposed to A/Sy-L/97 (Figure 3). This finding is discordant with the susceptibility of TH immunoreactive neurons (where A/NC-L/99 resulted in greater cell loss), and may be due to a different susceptible neuronal population (non TH immunoreactive).

**Figure 4:**
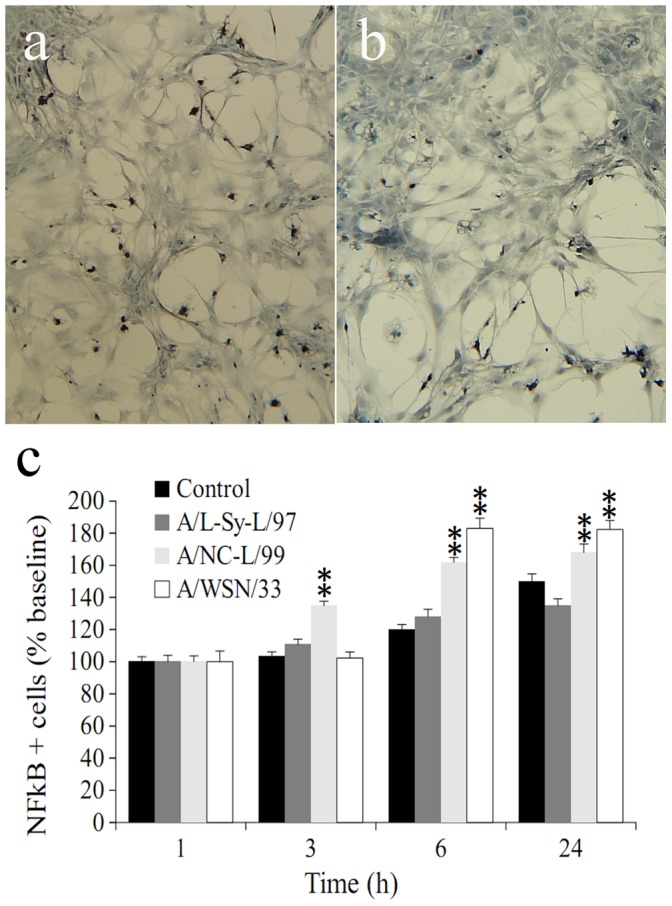
Influenza infection results in NFκB activation in vitro. Panels (a) and (b) show NFκB staining in primary cultures following inoculation of A/WSN/33 (a) or PBS solution (b). Cultures (11 day in vitro) were inoculated with PBS, A/WSN/33, A/NC-L/99 (H1N1), A/Sy-L/97 (H2N3) influenza strains as described in the text, fixed, immunostained for NFκB and neurons were counted at times specified in panel (c). Cell bars represent means of 6–8 independent experiments (two wells per experiment). Error bars are SEM. Comparisons were performed by two way ANOVA (treatment and time). ** p<0.001 compared to control (PBS solution).

We have previously described that programmed cell death of dopaminergic neurons in this culture system is mediated by NFkB (51), and others have shown that NFkB signaling is an early and necessary step in InfV-triggered apoptosis pathway [54]. NFkB immunostained neurons were distinguished from glial cells on morphological grounds (Figure 4A–B) and counted. As predicted, we found a marked increase in neuronal NFkB immunostaining between 3 and 6 hours post-infection with H1N1 strains, but not with H3N2 (Figure 4C).

### InfV Infection during Pregnancy Results in Behavioral Abnormalities in the Offspring

**Figure 5:**
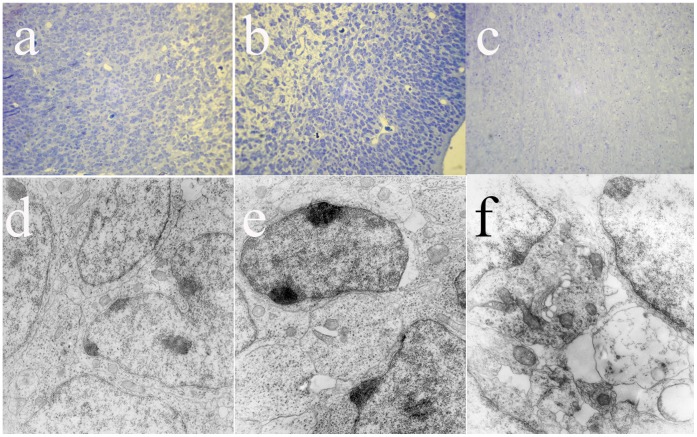
Morphological changes in embryos from mothers infected with influenza Virus strains sacrificed 3 days following inoculation of control solution (a,d), or infection with influenza A viral strains A/NC-L/99 (b,e) or A/WSN/33 (c,f) on pregnancy day 11. Top panels display low magnification (X200) of the area of the substantia nigra stained with Nissl. Bottom panels show electron micrographs (X24,000) obtained from the same specimens. Infection with influenza results in significant swelling of midbrain embryonic neurons. Arrowheads indicate vacuolar dilatations.

Pregnant (embryonic day 9–11) mice received intranasal infusions containing A/NC-L/99 (H1N1), A/WSN/33 (H1N1), or vehicle alone (control). Gross inspection of mesencephalon of mouse embryos 72 h after mothers were infected with InfV with Nissl staining at low magnification revealed dystrophic appearance of neurons, with significant cytosolic vacuolation and increased intercellular space, both of which were more apparent on offspring of mothers infected with A/WSN/33 strains (Figure 5A, 5C, 5E). Electron microscopy inspection of synaptic terminals revealed marked vacuolation of embryonic neurons (Figure 5B, 5D, 5F, arrowheads). This acute dystrophic changes at the ultrastructural level have not been described before. Both viral strains caused significant ultrastructural dystrophic alteration and loss of dopaminergic neurons in the mesencephalon of adult offspring of infected mothers (not shown). Of note, we were unable to detect viral antigens in brain by either RT PCR, indirect immunofluorescence or electron microscopy at any of the ages studied (three days after infection, and at post natal days 30 and 90) or with any of the viral strains used (not shown). A set of experiments were also performed with A/Sy-L/97 (H3N2) but, consistent with the *in vitro* findings, we found no seroconversions (that is, this strain did not cause infection to mice) and no data were obtained from offspring. Table 1 summarizes the number of animals infected, seroconverted and used for behavioral and histological studies, and Table 2 summarizes the results of the behavioral assessments. Exposure to a novel object was tested in a separate cohort of adult (90 days old) offspring of mothers inoculated with vehicle (n = 12, 6 males) or InfV A virus strains: A/NC-L/99 (n = 10, 6 males) or A/WSN/33 (n = 11, 6 males). Following birth, offspring of seroconverted mothers were allowed to grow in normal housing conditions. Behavioral testing included spontaneous activity, exploratory behavior in the open field, exposure to novel objects, and elevated cross maze either on post natal days 30–40 (pre-puberty) or post natal days 90–100 (adulthood). No differences between groups were observed in either diurnal or nocturnal spontaneous activity (data not shown).

**Table 1:** Summary of animals used in the in vivo experiments.

	dams		offspring	young offspring		adult offspring	
	*inoculated*	*seroconverted*	*Total*	*behavior*	*histology*	*behavior*	*histology*
Control	38	0	29	20	4	16	4
A/NC-L/99	24	17	23	13	4	9	4
A/WSN/33	39	22	34	25	4	18	4
A/Sy-L/97	9	0	12	0	0	0	0

**Table 2:** Behavioral assessments in offspring of infected mothers.

Test	Control	A/WSN/33	A/NC-L/99	ANOVA for Strain
***30 day old (young adults)***				
Open Field				
*rearings*	17.44+/−2.30	24.39+/−1.43	19.78+/−2.62	*F<1, d.f. = 2, p = n.s*
*trajectory (cm)*	972.86+/−184.67	1791+/−232.43	944.24+/−196.17	*H = 8.396, d.f. = 2, p<0.05*
*time in center (s)*	1.34+/−0.95	3.09+/−0.93	1.22+/−0.56	*H = 8.396, d.f. = 2, p<0.05*
Novel Object				
*Latency (s)*	448.05+/−40.47	284.64+/−38.21	318.69+/−59.1	*HF = 7.933, d.f. = 2, p<0.05*
*Time spent in contact (s)*	14.43+/−5.68	32.84+/−6.66	42.15+/−16.52	*H = 6.68, d.f. = 2, p<0.05*
Elevated plus maze				
*Time in open arms (s)*	10.05+/−0.99	15.36+/−1.93	8.46+/−1.86	*F = 4.55, d.f. = 2, p<0.05*
***90 day old (young adults)***				
Open Field				
*rearings*	14.13+/−2.22	28.56+/−2.80	26.00+/−3.29	*H = 11.19, d.f. = 2, p<0.01*
*trajectory (cm)*	879.79+/−174.46	1329.77+/−487.70	947.97+/−285.45	*F = 0.44, d.f. = 2, p = n.s.*
*time in center (s)*	0.70+/−0.53	27.50+/−8.16	30.53+/−14.53	*H = 11.29, d.f. = 2, p<0.01*
Novel Object				
*Latency (s)*	421.63+/−45.06	199.78+/−34.85	186.89+/−27.33	*F = 11.27, d.f. = 2, p<0.01*
*Time spent in contact (s)*	28.44+/−8.56	75.83+/−18.53	74.00+/−15.55	*H = 6.06, d.f. = 2, p<0.05*
Object Recognition				
*Δ (novel-familiar)*	5.45+/−1.63	0.80+/−3.68	−1.70+/−6.16	*F = 19.30, d.f. = 2, p<0.01*
Elevated plus maze				
*Time in open arms (s)*	6.13+/−0.97	14.11+/−1.67	5.13+/−1.34	*F = 12.14, d.f. = 2, p<0.01*

Means are provided for for exploratory behavior, rearing and and anxiety levels in the Open Field, exploratory behavior of a novel object, and performance on an elevated cross maze test of young (post natal day 30) and adult (post natal day 90) offspring of mothers inoculated with control solution (PBS) or infected with influenza A strains A/WSN/33 or A/NC-L/99. A separate cohort of adult offspring was tested for an object recognition task; time spent exploring the familiar and non-familiar object was computed and the difference (Δ) is reported. Values represent means ± SEM. Bold values in grayed cells represent p<0.01 compared to control. Italic values in grayed cells represent p<0.05 compared to control. Statistics represent ANOVA followed by Kruskal Wallis post hoc comparisons for means.

Open field behavior in young mice revealed a significant effect of prenatal exposure to InfV A, whether considering total trajectories, number of rearings, or time spent in the central area. Young offspring of mothers infected with A/WSN/33 had longer trajectories, higher number of rearings and spent more time in the central area of the open field than animals exposed to A/NC-L/99 or controls. The latter two were not distinguishable on this test. Likewise, adult offspring from mothers exposed to A/NC-L/99 or A/WSN/33 had significantly more rearings and spent more time in the central area than offspring of control mothers, but the increase in total trajectory did not reach significance.

When a novel object was introduced to young mice, exposure to InfV A during pregnancy resulted in significantly reduced latency to the first contact with the novel object, and more time spent in contact with it during the session. However, even though the mean values for offspring of mothers infected with A/NC-L/99 were indistinguishable from those of mothers infected with A/WSN/33, only the latter were significantly different from control offspring, suggesting a type 2 error. Indeed, when adult mice were exposed to the novel object offspring from mothers exposed to both strains showed significantly reduced latency and increased contact with the novel object. During the object recognition task exploration times during the first session (T1) were unaffected by prenatal exposure, even though offspring of mothers infected InfV A strains showed a trend towards longer exploration times. On the other hand, exploration times of the familiar and non-familiar objects during T2 revealed a significant effect of prenatal exposure to InfV A such that offspring of control mothers spent significantly more time exploring the novel, non-familiar object while no differences were observed in offspring of mothers infected with A/NC-L/99 or A/WSN/33, suggesting working memory impairment.

In the elevated cross maze both young and adult offspring from A/WSN/33 infected mothers spent significantly more time in the open arms, whereas offspring of A/NC-L/99 infected mothers were undistinguishable from controls.

Multivariate analysis of the behavioral data by discriminant analysis revealed that the net effect of prenatal infection with InfV virus on behavior could be described by two statistically significant discriminant functions accounting for more than 90% of the variance (*Chi square* = 24.208, *d.f.* = 12, *p*<0.05). These two functions allowed correct classification of 100% of the offspring of mothers infected with A/WSN/33, and of 83% of those of mothers infected with A/NC-L/99. The reminder 17% were indistinguishable from controls (Table 3).

**Table 3:** Canonical Discriminant Analysis of Behavioral Data.

Eigenvalues				
Discriminant Function	Eigenvalue	% of variance	Cumulative %	Canonical Correlation
1	63.842	91.9	91.9	0.992
2	5.632	8.1	100	0.922
**Wilks’ Lambda**				
**Test of Functions**	**Wilks’ Lambda**	**Chi-square**	**d.f.**	**significance**
1 through 2	0.02	22	36.383	0.28
2	0.151	11.351	10	0.331
**Functions at Group Centroids**				
**Influenza Strain**	**Discriminant Function**			
	1	2		
Control	−4.595	2.470		
A/NC-L/99	8.020	−0.475		
A/WSN/33	−8.382	−3.167		
**Classification Results**				
**Influenza Strain**	**Predicted Group Membership**			
	**Control**	**A/NC-L/99**	**A/WSN/33**	
Control	100	0	0	
A/NC-L/99	0	100	0	
A/WSN/33	0	0	100	

Two significant functions were identified which allowed correct classification of all animals, suggesting that neuroadapted (A/WSN/33) and respiratory (A/NC-L/99) strains of H1N1 influenza result in significant differences in severity of abnormal behavior in the offspring of mothers infected during pregnancy.

In summary, maternal infection with both strains resulted in abnormal behavior in the offspring, including time spent in the open arm of the elevated plus maze, changes in exploratory behavior, reduced latency to contact and increased time in contact with a novel object, and working memory impairment when compared to offspring of mothers exposed to vehicle. Additionally, the behavioral alterations appear earlier in the offspring of mothers infected with A/WSN/33.

### InfV Infection during Pregnancy Results in Selective Loss of Dopaminergic Neurons in the Adult Offspring

**Figure 6:**
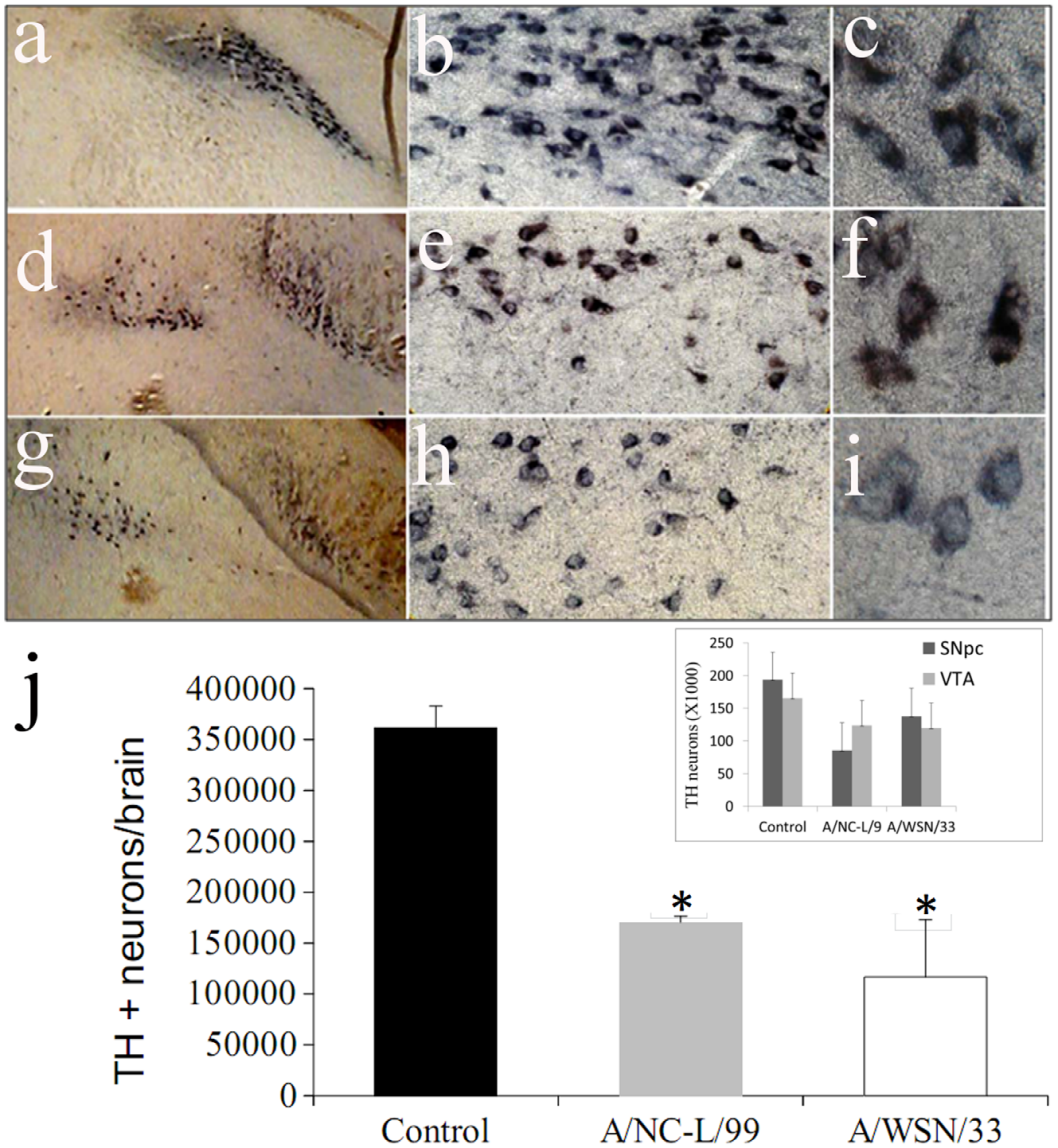
Midbrain dopaminergic nuclei in the adult (p90) offspring of mothers inoculated during pregnancy with control solution (a,b,c), or infected with influenza strains A/NC-L/99 (d,e,f) or A/WSN/33 (g,h,i) during pregnancy day 11. From left to right, panels show increasing magnification of tyrosine hydroxilase immunostaining (100X, 400X, 1000X). There is a clear loss of stained neurons in the exposed animals, and at higher magnification a dystrophic appearance of surviving neurons is evident. Panel (j) displays mean ± SEM of stereological counts of dopaminergic (TH positive) neurons throughout the entire brainstem (n = 4/group). * p<0.05 compared to control. The insert displays the results discriminating between substantia nigra pars compacta (SNpc) and ventral tegmental area (VTA) in a separate set of 4 animals for each viral strain.

**Figure 7:**
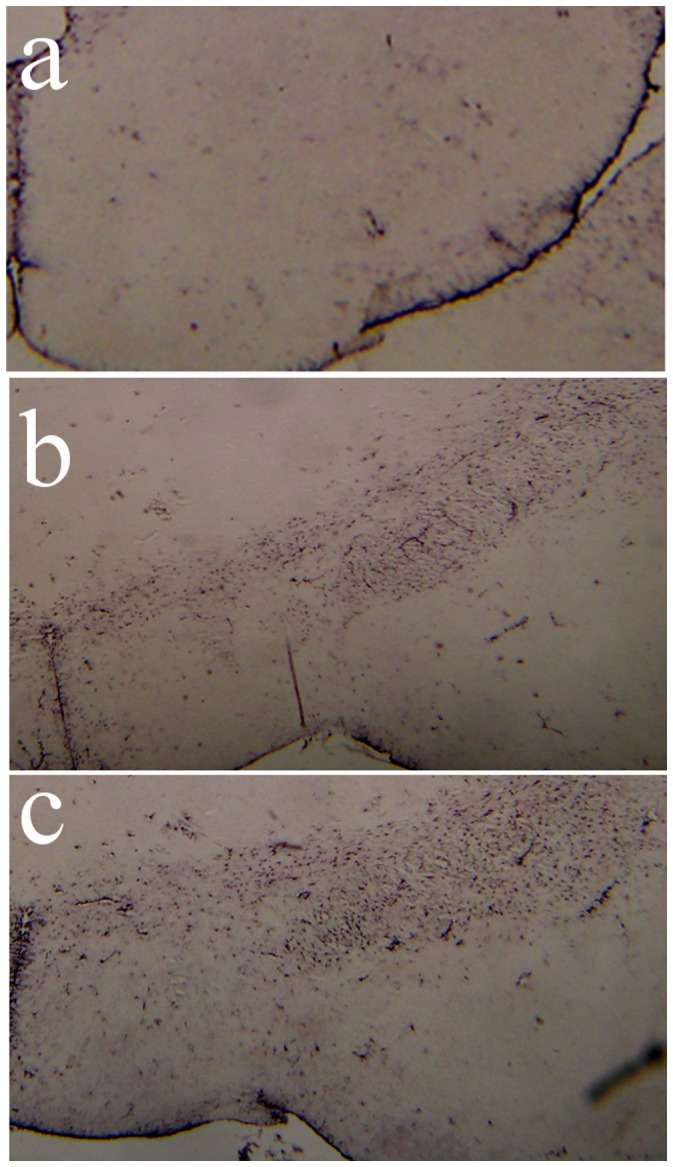
Midbrain gliosis in the adult (p90) offspring of mothers inoculated during pregnancy with control solution (a), or infected with influenza strains A/NC-L/99 (b) or A/WSN/33 (c) during pregnancy day 11. Sections consecutive to the ones used for Figure 6 were stained for activated glia with antibodies against glial fibrilary acidic protein (GFAP) and photographed at low magnification (100X). There is a clear increase in reactive astrocytes in the two experimental conditions, when compared to controls.

Histopathological abnormalities in the offspring of mice infected with InfV strains during pregnancy was carried out in two sets of samples obtained at p30 (n = 4 per group), and at p90 (n = 4 per group). Both viral strains caused significant dystrophic alteration and loss of dopaminergic neurons in the mesencephalon of adult (post-natal day 90) offspring of infected mothers (Figure 6A–I). Quantitative stereology demonstrated a 30% loss of tyrosine hydroxylase immunoreactive neurons in adult offspring of A/NC-L/99 infected mothers, and a 50% loss in those of mothers infected with A/WSN/33 (Figure 6J). When the effects of viral strains were discriminated between TH positive neurons in the substantia nigra pars compacta (SNpc) vs. the ventral tegmental are (VTA) in a separate set of experiments, similar reductions were observed in both nuclei (Figure 6J, inset). In adolescent offspring (post natal day 30) the effects were virtually identical (not shown). A corresponding increase in glial acidic fibrilary protein (GFAP) immunostaining indicating gliosis was observed in the same brain region of the adult offspring of mice infected with InfV A strains during pregnancy (Figure 7). This increase was again most marked for animals born to mothers infected with WSN/33 (Figure 7C), and intermediate for those born to mothers infected with A/NC-L/99 InfV A virus (Figure 7B).

## Discussion

The three major findings of the present work provide evidence in favor of the hypotheses tested. First, we found that circulating strains of InfV infect cultured mesencephalic neurons resulting in selective loss of dopaminergic neurons; this effect was dependent on the antigenic configuration of the strain, it was preceded by NFkB activation and mediated by apoptosis. Both H1N1 strains of InfV had the greatest affinity for dopaminergic neurons, whereas an H3N2 strain induced apoptosis preferentially in other cell types, and did not result in NFkB activation.

Second, following maternal infection with H1N1 (but not H3N2) InfV strains we found a persistent and a selective loss of dopaminergic neurons in substantia nigra pars compacta and ventral tegmental area of the offspring (Figure 6J, inset). Perhaps not surprisingly, loss of dopaminergic neurons was more pronounced in the adult offspring of mothers infected with the neuroadapted A/WSN/33 than with the respiratory strain A/NC-L/99. Likewise, neuroadapted A/WSN/33 was associated with greater behavioral impairment than A/NC-L/99.

Third, offspring of mother infected with both InfV strains showed marked behavioral abnormalities in exploration, anxiety and working memory. Additionally, behavioral alterations emerge in different neurodevelopmental stages depending on the InfV strain, appearing in adult life in offspring of mother infected with A/NC-L/99.

Could the specific findings with each viral strain could perhaps be explained by differences in LD50 or TCID50, rather than because of antigen configurations? We specifically assessed this question and found that TCID50 of both H1N1 strains in brain primary cultures was similar, but one order of magnitude higher than the TCID50 for the H3N2 isolate. Yet, the H3N2 strain caused more apoptosis than either H1N1 strain, indicating that neurotoxicity is not limited by TCID50. On the other hand, the effect of both H1N1 strains was selectively higher on dopaminergic neurons, again indicating specificity of this effect. In vivo, all three strains resulted in effective infection judged by increased lung weight, such that A/NC-L/99 =  A/WSN/33> A/Sy-L/97. Indeed, no seroconversions were found after infection of the dams with the latter strain and thus no further studies were carried out in their offspring. In summary, differences in infectivity between strains do not explain the specificity of their effects on different neuronal populations (i.e., dopaminergic vs non-dopaminergic) *in vitro*. Rather, H1N1 strains induced toxicity to dopaminergic neurons at viral loads in which the H3N2 strain caused significant apoptosis in other neuronal populations but not in dopaminergic neurons. On the other hand, the lower infectivity of A/Sy-L/97 in mouse explains its lack of effects *in vivo*. Yet, adapted and non-adapted H1N1 strains were equally infective (by ID50) and resulted in comparable neurodevelopmental damage to the offspring. Lastly, differences in LD50 or TCID50 between H1N1 and H3N2 strains (higher or lower dose) could lead to differences in innate cell activation upon infection, chemokine or cytokine expression leading to differences in the magnitude of the innate and adaptive immune response and differences in the pathogenic potential of the virus or the spread of the virus and the infection beyond the target organ.

### Influenza Virus, Schizophrenia and Parkinsonism

Schizophrenia is a heterogeneous disorder with genetic, epigenetic and environmental factors contributing to its causation [6], [11], [43], [55]. Yet, the precise way in which such factors contribute to a common pathophysiology giving rise to a recognizable syndrome remains far from established. Evidence of abnormal development in schizophrenia includes increased incidence of craniofacial asymmetries, dermatoglyphic irregularities, and disturbed neuronal migration, which point to a noxius process acting between the first and second trimester of pregnancy [55]. A relationship between viral infections and the onset of psychosis has been suspected for a long time because schizophrenia-like psychoses occur after InfV pandemics. Even though the extent of the association remains controversial, some patients with schizophrenia show increased proinflammatory cytokines, acute phase proteins, and TH-2 activity [56]. Yet again, no clear mechanism for the contribution of these environmental events to inherited (genetic) risk of disease has been established, even assuming that infections could either initiate schizophrenia by direct brain lesion or by triggering an autoimmune response during the neurodevelopmental period on a genetically susceptible brain [55]. Genetic association findings have contributed somewhat to clarification of the mechanism, since genes thought to contribute risk are related to cell division and differentiation, neuronal cell adhesion, neurotransmission and neuroplasticity, oligodendrocyte function, and immune responses to pathogens implicated in the disease [57]. Most notably, Disrupted in schizophrenia 1 (DISC1) controls the microtubule network that is used by viruses as a route to the nucleus, and Neuregulin 1 activates ERBB receptors releasing a factor, EBP1, known to inhibit the InfV transcriptase [57]. Several other genes may affect pathogen virulence, while the pathogens in turn may affect expression of genes and processes underlying schizophrenia; thus, genetic association may be conditioned by the presence of the pathogen [57].

In 1916, an epidemic of encephalitis lethargica suggested an association between viral infections, parkinsonism and psychosis, and a recent case series confirmed an encephalitis of the midbrain and basal ganglia [58], even though a role for InfV in this pathology remains controversial. In any case, once InfV A strains adapt to the central nervous system, they tend to show great affinity for dopaminergic neurons in the substantia nigra both in human cases and experimental models, and on this basis it has been proposed that InfV may play a role in the etiology of Parkinson’s disease [59].

Thus, the net effect of InfV infections on brain pathology may depend on genetic susceptibility, antigenic strain characteristics, and neurodevelopmental stage. Our data support the view that in susceptible individuals, prenatal infection may result in selective destruction of key dopaminergic projections increasing the risk of neuropsychiatric disorders.

### Mechanism of Tissue Selectivity of Influenza

Extracellular cleavage of the hemagglutinin by host trypsin-like proteases is a prerequisite for the infectivity and pathogenicity of human InfV A, and tissue specific infectivity is largely dependent on the availability of proteases [60]. On the other hand, neuraminidases are incorporated and detected inside neurons with a diffuse cytoplasmic and nuclear distribution, followed later by concentration in dendrites [61]. InfV bind to human and murine PTX3, a protein that activates complement and facilitates pathogen recognition by macrophages [62]. PTX3 is rapidly induced following InfV infection and therapeutic treatment of mice with human PTX3 promotes survival and reduces InfV load [62]. It is worth pointing out that PTX3 is a unique transcription factor marking the mesencephalic dopaminergic neurons at the exclusion of other dopaminergic neurons, and it may be involved in developmental determination of this neuronal lineage [63].

### Selectivity of Neuroadapted H1N1 Strains

Neurovirulence in mice is a unique property of some InfV strains. We included a neurovirulent strain (A/WSN/33) as a positive control in our experiments. A brief review of the published effects of direct infection with this InfV strain in brain is warranted. Indeed, A/WSN/33 in culture strictly infect neurons [26], and have highest affinity for TH-positive neurons in substantia nigra [26]. Furthermore, intracerebral inoculation of A/WSN/33 in mice results in early infection of neurons in circumventricular regions, cerebral cortices, substantia nigra pars compacta and the vental tegmental area, but later accumulates in substantia nigra pars compacta and hippocampus [24], [64], [65]. Following direct injection of A/WSN/33 into the olfactory bulb, InfV-infected neurons appear quickly in the anterior olfactory nucleus, habenular, paraventricular thalamic, ventral tegmental area, amygdala and the pyramidal layer of the hippocampus [27], [66]–[70].

Mice survive and the viral infection is cleared from the brain, but a variety of neuronal changes occur over a period of weeks. Infected neurons up regulate Fas ligand molecules leading to activation of JNK signal transduction pathway followed by DNA fragmentation and activation of caspase-3 [68], [69]. Also, interleukin 1b and tumor necrosis factor α expression increase in infected neurons [71]. Eventually infected neurons undergo apoptosis. In the habenular and paraventricular thalamic areas, infection results in an almost total loss of neurons.

Viral gene products are eliminated from brainstem dopaminergic neurons by a mechanism dependent on Transporter associated with Antigen Presentation 1 (TAP1) [66]. Activated microglial cells appear throughout the brain eventually clearing apoptotic bodies [27], [28], [68], [70], [72]. However, genomes of A/WSN/33 persist in the brains of immunodefective TAP1 mutant mice [23], [73], and knock out of IFN-γ receptor, iNOS, or TAP1 result in viral persistence in the olfactory bulb [74].

A/WSN/33 injection resulted in behavioral changes months after infection, including increased exploration in the open arms of an elevated plus-maze [75], impaired spatial learning in the Morris water maze test [75], increase in non-rapid eye movement sleep [76] and decrease in rapid eye movement sleep [76]. Abnormalities in working memory and exploratory behavior are more prominent in immunodeficient mice [77]. These effects of InfV interact with genes associated with risk of schizophrenia. In the medial prefrontal cortices reduced levels of neregulin 1 transcripts were observed [77]. Likewise, elevated transcriptional activity of regulator of G-protein signaling 4 (RGS4) and calcium/calmodulin-dependent protein kinase IIa, were found in the amygdala, hypothalamus and cerebellum [75]. Interleukin-1β and TNF-α increases may explain the changes in sleep [76], but a contribution of nitric oxide synthase induction has also been noted [18], [76].

Lastly, after intranasal inoculation of A/WSN/33 invasion of brain also occurs, once again with greatest affinity for catecholaminergic neurons [78].

In addition to direct neuronal infection and apoptosis, several other mechanisms have been invoked that could result in neuronal damage. Secondary production of autoantibodies against neuronal populations in hippocampus, cerebral cortex and cerebellum, also may occur after infection with H1N1 InfV viruses, including A/WSN/33 [20]. On the other hand, neuronal death may be the consequence of excitotoxicity, since turnover rate of glutamate in brain, increases in the InfV-associated encephalopathy [79]. On the other hand, it A/WSN/33 experimental infection activates the entire kynurenine pathway, which should result in antagonism on the NMDA receptor [80] with a variety of effects on neuronal survival that would largely depend on the developmental stage of the brain, with apoptosis being the predominant effect during early development [81].

### Neurotropism of Respiratory InfV Strains

Experimental infection with InfV with human virus isolates without adaptation to an animal host has been also shown to result in neuropathological outcomes and changes in brain function associated with areas of virus replication in neurons [82]. Our data obtained with common circulating strains of respiratory influenza confirm this findings when antigen configuration is H1N1.

### Effects on the Offspring after Maternal Infections

In brains exposed prenatally to A/WSN/33, glial fibrillary acidic protein (GFAP), an important marker of gliosis, neuron migration, and reactive injury increases in cortical and hippocampal cells; GFAP-positive cells have ‘hypertrophy’ and more stellate morphology [18]. These results implicate a significant role of prenatal human InfV viral infection on subsequent gliosis, which persists throughout brain development in mice from birth to adolescence [19]. Yet, in spite of our extensive efforts we have been unable to detect viral antigens after birth (specifically, we tested at P30–40 y P90–100) either by PCR or immunohistochemistry. Indeed, we exhausted our methodological options in the hope to reproduce the findings of Aronsson et al [23]. On the other hand, our negative finding is consistent with the report by Shi et al [10] and few others. Also in agreement with the report by Shi [10] we did not detect viral antigen three days after maternal infection at embryonic day 12 to 14 (not shown). Thus, our findings support the interpretation that the inflammatory processes within the central nervous system may be mediated at least in part by secondary immune responses triggered or ongoing after the viral cycle is completed [10]. In addition, prenatally exposed brains showed significant reductions in reelin-positive cell counts in layer I of neocortex and other cortical and hippocampal layers, as well as decreases in neocortical and hippocampal thickness [17].

Exposure to the lipopolysaccharide during the critical developmental window in rats (embryonic day 10.5), leads to the birth of animals with fewer than normal dopaminergic neurons, reduction in striatal dopamine, and increased TNF-α [83], [84]. This dopaminergic neuron loss is apparently permanent and increases with age [84]. Furthermore, animals exposed to prenatal lipopolysaccharide have increased susceptibility to environmental toxins postnatally [84].

In summary, there is at least one strain of circulating InfV (not pandemic) in Argentina able to selectively decrease of dopaminérgic neurons in the offspring of dams infected during a critical developmental period. Selective loss of dopaminergic neurons follows activation of NFkB and apoptosis, and results in profound behavioral abnormalities when the animals reach adulthood.
